# Experience With Dexmedetomidine Use in the Treatment of Dysautonomic Crisis in Familial Dysautonomia: An Off-Label Use

**DOI:** 10.7759/cureus.29988

**Published:** 2022-10-06

**Authors:** Ananta Subedi, Rakshya Sharma, Ishan Lalani

**Affiliations:** 1 Internal Medicine, Avera McKennan Hospital & University Health Center, Sioux Falls, USA; 2 Pulmonary and Critical Care Medicine, University of Alabama at Birmingham, Birmingham, USA

**Keywords:** off-label drug use, dexmedetomidine, autonomic crisis, dysautonomic crisis, familial dysautonomia

## Abstract

Familial dysautonomia is a rare genetic neurodevelopmental disorder characterized by episodes of hyperautonomic state known as dysautonomic crises. The features of dysautonomic crises are hypertension, tachycardia, vomiting, sweating, flushing, and behavioral changes. The etiology of such crises is supposed to be a consequence of the inability to control sympathetic overflow due to damage to the afferent neurons carrying baroreceptor inputs to the central nervous system.

A 19-year-old male with a known history of familial dysautonomia and frequent dysautonomic crises presented to the Emergency Department with intractable nausea and vomiting for six hours. He was hypertensive and tachycardic on presentation. The patient had tried oral labetalol and clonidine at home with no improvement. In the emergency room, the patient received intravenous labetalol, diazepam, and clonidine which were ineffective. He was then treated with intravenous dexmedetomidine, and his symptoms resolved within a few hours. The patient was discharged home on the same day.

The mainstay of treatment for dysautonomic crises is benzodiazepines and clonidine. The use of these treatment modalities has its challenges. Here, we present a case of a dysautonomic crisis that was resistant to the conventional treatment, treated safely and successfully with dexmedetomidine.

## Introduction

Familial dysautonomia is a rare neurodevelopmental genetic disorder characterized by episodes of hyperautonomic state known as dysautonomic crises. They are recurrent, refractory, and paroxysmal episodes of hypertension, tachycardia, sweating, flushing, vomiting, swallowing dysfunction, and speech dysfunction [1]. The mainstay of treatment for such dysautonomic crises has been benzodiazepines and clonidine [2]. Cases refractory to the treatment with benzodiazepines and clonidine have also been treated with carbidopa [3]. There have been a few case reports of successful treatment of such crises using dexmedetomidine. Here, we present a case of dysautonomic crisis which was effectively treated with dexmedetomidine after no response to the treatment with labetalol, diazepam, and clonidine. In a rare disease like familial dysautonomia, which has limited treatment options, awareness about successful treatment of the refractory cases with an agent such as dexmedetomidine will be beneficial to prevent significant morbidity [4].

## Case presentation

A 19-year-old male with a history of familial dysautonomia with frequent autonomic crises was brought to the Emergency Department by his parents for intractable nausea and vomiting for six hours. On average, the patient had two to three episodes of dysautonomic crises per week which usually resolved with oral labetalol and clonidine. The patient was also restless and hypertensive. His systolic blood pressure was 210 mmHg at home. He was also warm and felt febrile. The patient usually had similar symptoms when he had episodes of dysautonomic crises. He was treated with three doses of oral labetalol and clonidine at home with no improvement. He did not have a history of sick contacts. He denied having any headaches, chills, cough, shortness of breath, abdominal pain, rash, diarrhea, constipation, hematuria, or dysuria. He did not have any history of smoking, alcohol use, or drug abuse. The patient stated that he had undergone a comprehensive outpatient workup for his episodes of hypertension and common etiologies were ruled out. The episodes had been attributed to his known diagnosis of familial dysautonomia.

On presentation, he was hypertensive and tachycardic. His initial vitals revealed a blood pressure of 206/158 mmHg, His heart rate was 109 beats per minute, his temperature was 97.2°F, and his respiratory rate was 16 breaths per minute. On physical examination, he had dry mucous membranes and dry eyes. He had bilateral clear breath sounds on chest auscultation. On cardiac auscultation, he had tachycardia but no murmurs. The abdomen was soft, non-distended, and non-tender. The rest of the physical examination was non-contributory. Initial bloodwork that included a complete blood count (CBC) and comprehensive metabolic panel (CMP) revealed a white blood cell (WBC) count of 14,800 but was otherwise unremarkable. Serum troponin was negative. Procalcitonin was within normal range and blood cultures were negative. A chest X-ray was done which showed no acute cardiac or pulmonary findings. An electrocardiogram was done which showed a normal sinus rhythm.

He was initially treated with Intravenous (IV) labetalol 20 mg, IV diazepam 10 mg, and oral clonidine 0.1 mg. He also received IV ondansetron 4 mg and oral acetaminophen. Repeat blood pressure in 10 minutes was unchanged. Then, he received IV labetalol 40 mg. His symptoms did not improve. His blood pressure after one hour was 201/161 mmHg. He was then admitted to the Intensive Care Unit and IV dexmedetomidine was started at the rate of 0.4 µg/kg/hour. This was up titrated by 0.1 µg/kg/hour every 30 minutes until the desired blood pressure was reached. The repeat blood pressure in the next hour was 171/132 mmHg. Within three hours, his blood pressure normalized and his symptoms resolved. Dexmedetomidine was then titrated down by 0.2 µg/kg/hour every 15 minutes and stopped. He was monitored for another six hours without any evidence of relapse. He was then discharged home.

## Discussion

Familial dysautonomia is a rare autosomal recessive neurodevelopmental disorder mostly seen among Ashkenazi Jews [4]. It is caused by a point mutation in the *I-k B kinase-associated protein* (*IKAP*) gene. Deficiency of the *IKAP *gene results in impaired development of sensory neurons. This causes impaired feedback from arterial baroreceptors and uncontrolled sympathetic outflow resulting in episodes of hypertension and tachycardia. This is usually triggered by emotional and physical stress. These episodes are known as dysautonomic crises which are recurrent, refractory, and paroxysmal episodes of catecholamine surge resulting in hypertension, tachycardia, sweating, flushing, vomiting, swallowing dysfunction, and speech dysfunction [1]. During such a catecholamine surge, dopamine is also released into the circulation along with epinephrine and norepinephrine. The increase in plasma dopamine levels activates D2 and D3 receptors in the chemoreceptor trigger zone of the area postrema which causes vomiting [5].

Dysautonomic crises are treated with benzodiazepines and clonidine. Benzodiazepines bind to gamma-aminobutyric acid (GABA) receptors and facilitate inhibitory neurotransmitters. In familial dysautonomia, it seems to help with dysautonomic symptoms. Clonidine is a central alpha-adrenergic agonist which seems to suppress the peripheral release of norepinephrine [5]. Our patient received labetalol as well which is a beta-blocker. Beta-blockers are competitive antagonists of catecholamines on adrenergic beta receptors which reduce blood pressure and heart rate. The use of these first-line agents at home and in the Emergency Department was ineffective for our patient. Carbidopa, which inhibits dopamine synthesis, has also been used in some cases for dysautonomic crises [3]. Dexmedetomidine is a centrally acting α2 adrenergic agonist which works as a sedative and anesthetic by acting on the G-protein in the brain and spinal cord. This results in the inhibition of norepinephrine release and reduction of sympathetic activity, resulting in hypotension, bradycardia, analgesia, and decreased bowel motility [4].

Controlled studies on the effectiveness of any treatment for dysautonomic crises including the first-line agents are lacking. There is no specific guideline for the management of dysautonomic crises. Regardless, the first-line agents used for dysautonomic crises have their limitations. Benzodiazepines can cause sedation and suppression of respiratory drive and are very addictive. Tolerance and dependence develop rapidly which limits its usefulness in patients who have frequent episodes of dysautonomic crises. Clonidine can cause sedation, fatigue, and hypotension and can cause rebound hypertension which also makes it less desirable for use in frequent dysautonomic crises [6]. Carbidopa can be an effective treatment for vomiting during attacks of dysautonomic crises but its effects on blood pressure are not very clear [3]. There have been a few anecdotal cases where the dysautonomic crisis was successfully treated with dexmedetomidine. There is one retrospective study in which electronic medical records of nine patients with dysautonomic crises who were successfully treated with dexmedetomidine were reviewed. The nine patients had 14 hospital admissions, and in 10 out of the 14 admissions, there was a decrease in blood pressure and heart rate after dexmedetomidine use [7]. There are no prospective studies evaluating the use of dexmedetomidine in dysautonomic crises. Further, familial dysautonomia is a rare disease, and finding enough subjects to conduct a study with sufficient power is difficult. Hence, our presented case can serve as a tool to indicate that dexmedetomidine can be a safe and effective treatment of refractory dysautonomic crises. Moreover, our patient could be discharged within a few hours of admission thus preventing longer hospital stays and significant morbidity.

## Conclusions

Familial dysautonomia usually presents with episodes of dysautonomic crises which are treated with benzodiazepines and clonidine. In case of refractory dysautonomic crises, where first-line treatment is ineffective, dexmedetomidine can be a safe, effective, and efficient treatment. However, further studies to evaluate the role of dexmedetomidine in dysautonomic crises are needed.
